# Baby Farming

**Published:** 1900-03-31

**Authors:** Ellen B. Kingsford, Blanche Wright

**Affiliations:** secretary, Home for Homless Babies, Hersham; matron, Home for Homless Babies, Hersham


### BABY FARMING.

Miss Ellen B. Kingsford, the secretary, and Miss Blanche Wright, the matron of the Home for Homeless Babies, Hersham, write: In reference to a recent annotation in The Hospital headed "Baby Farming," we should be glad to say a few words. The enclosed papers will show that we are engaged in this "very questionable business." Whilst cordially endorsing your strictures on the Act for exempting those who deal with one child only, we would submit that to include under it "institutions" established for the care of infants, and conducted "in good faith" and "for religious and charitable purposes," would only place an additional obstacle in the way of those who might otherwise be willing to do something to save at least some of the nameless babies from the fate of Williams' victim. In large towns the objection to being within the meaning of the Act might not be so strong, for there an officer—often a lady—with special qualifications is told off for the work entailed by the Infant Life Protection Act. In country villages this work often devolves upon the relieving officer, who probably knows nothing whatever of the requirements of infants, even as to cubic space, and whose periodical visits would be both uncalled for and useless, as the inspection of charitable institutions can scarcely come within the limits of his duties. Baby farming is a work in which it is amazingly difficult to interest the public. On looking through our list of subscribers and donors we find only two donors who, as far as we know, support the home on its own merits. The rest are our personal acquaintances, or those who have business connections with the home.

*_** Doubtless it is true that in many country districts the relieving officer may have been "told off" to do the work. But the Act allows either male or female inspectors to be appointed. The present condition of affairs is largely due to the fact that baby farming as dealt with by the Act still remains, as we have described it, a "very questionable business," the endeavour of those who framed the Act having been to exclude from its provisions as many as possible of those cases in which the care of infants has been undertaken from honest motives. If those who had to do with the passing of the Act could have brought under its provisions all who not being the parents took charge of infants for payment, including charitable institutions and even relatives, the stigma of inspection would have been removed, and there would have been no greater shock to respectability in the call of the inspector to inquire how the stranger child was getting on than there is in his or her calling to inquire after the state of the drains. The taint of disrepute which hangs over the proceedings under this Act is entirely the result of its application being so arbitrarily restricted. We do not suggest that a charitable institution should be placed under the control of the inspectors of the present Act, but we do hold that some guarantee in the form of inspection should be given to the public in the case of every infant which is separated from its parent and taken in to nurse for payment, whether this is done by a relative, a charitable institution, a perfectly respectable woman who brings up a single nurse child as one of her own family, or by one of those wretches who take in these unfortunate infants for the sake of getting rid of them. If this were done there would be some hope of stopping the machinations of the latter, and we cannot believe that the slightest injury would be inflicted upon those charitable ladies who, as in the case of our correspondents, give themselves up to what we cannot but regard as one of the kindest of charities. Unfortunately, as the result of the exemptions contained in the Act, its effect has been that instead of the "baby-farmer" (using the term in its bad sense) taking in a lot of children and merely letting the weakly ones die, they have been driven to get rid of them more quickly, seeing that if they have more than one at a time they must report and be inspected, and this has led to murder.—Ed. *T.H.*

